# Anti-Cancer and Anti-Proliferative Potential of Cannabidiol: A Cellular and Molecular Perspective

**DOI:** 10.3390/ijms25115659

**Published:** 2024-05-23

**Authors:** Manamele Dannies Mashabela, Abidemi Paul Kappo

**Affiliations:** Department of Biochemistry, Faculty of Science, University of Johannesburg, Auckland Park Kingsway, P.O. Box 524, Johannesburg 2006, South Africa; akappo@uj.ac.za

**Keywords:** apoptosis, autophagy, cancer, cannabinoids, cannabis, CBD

## Abstract

Cannabinoids, the bioactive compounds found in *Cannabis sativa*, have been used for medicinal purposes for centuries, with early discoveries dating back to the BC era (BCE). However, the increased recreational use of cannabis has led to a negative perception of its medicinal and food applications, resulting in legal restrictions in many regions worldwide. Recently, cannabinoids, notably Δ^9^-tetrahydrocannabinol (THC) and cannabidiol (CBD), have gained renewed interest in the medical field due to their anti-cancer properties. These properties include the inhibition of tumour growth and cell invasion, anti-inflammatory effects, and the induction of autophagy and apoptosis. As a result, the use of cannabinoids to treat chemotherapy-associated side effects, like nausea, vomiting, and pain, has increased, and there have been suggestions to implement the large-scale use of cannabinoids in cancer therapy. However, these compounds’ cellular and molecular mechanisms of action still need to be fully understood. This review explores the recent evidence of CBD’s efficacy as an anti-cancer agent, which is of interest due to its non-psychoactive properties. The current review will also provide an understanding of CBD’s common cellular and molecular mechanisms in different cancers. Studies have shown that CBD’s anti-cancer activity can be receptor-dependent (CB1, CB2, TRPV, and PPARs) or receptor-independent and can be induced through molecular mechanisms, such as ceramide biosynthesis, the induction of ER stress, and subsequent autophagy and apoptosis. It is projected that these molecular mechanisms will form the basis for the therapeutic applications of CBD. Therefore, it is essential to understand these mechanisms for developing and optimizing pre-clinical CBD-based therapies.

## 1. Introduction

The medicinal properties of cannabis (*Cannabis sativa*), also known as marijuana, have been well documented, with the earliest records dating back to texts on Ayurvedic medicine and the *Shen Nung Pen Ts’ao Ching*, the earliest-known pharmacopeia [1,2]. Archaeological investigations have provided further evidence of the historical use of cannabis for religious, spiritual, food, and textile purposes from as early as the third millennium BC [2]. At the same time, herbal and medicinal applications can be traced back as early as 500 BC [1]. The adoption of cannabis in Western medicine emerged in the mid-1500s and 1600s in South and North America, respectively. At the same time, an introduction to the European medical fraternity came through groundbreaking work on Indian hemp by William O’Shaughnessy in 1839 [3,4]. Wood and colleagues first isolated a compound named cannabinol from Indian hemp in 1899, followed by the chemical characterization of cannabinoids, including Δ^9^-tetrahydrocannabinol (THC) and cannabidiol (CBD), in the 1960s [4,5]. However, the popularised psychotic effects of cannabis and its widespread distribution as an illicit drug led to the marginalization and criminalization of this natural evergreen in the 20th century, ultimately impeding medicinal research on the previously documented medicinal properties of this plant [2,6].

**Figure 1:**
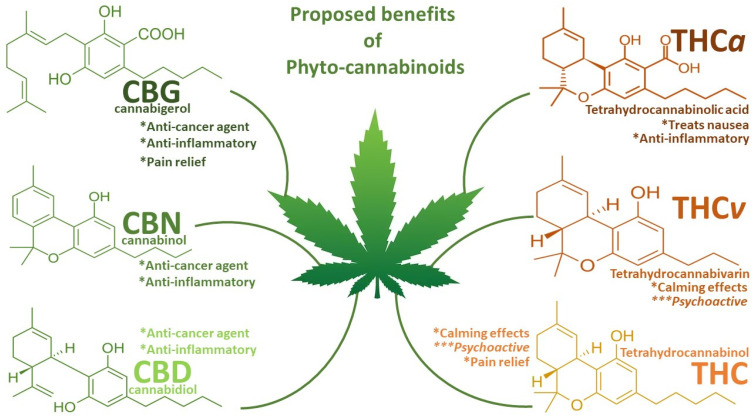
Summary diagram of the proposed benefits of phytocannabinoids. Special attention is given to the anti-cancer and anti-inflammatory capabilities of CBG, CBN, and CBD and their lack of calming or psychoactive effects, which has justified their pursuit in medical research, particularly on CBD over the THC and THC derivatives. * = beneficial effects; *** = negative effects.

Cannabinoids (CBNs) are a large group of naturally occurring compounds; this diverse group of approximately 120 cannabinoids is classified into three major categories: phytocannabinoids (found in *Cannabis sativa*), endocannabinoids (physiologically produced in humans), and synthetic cannabinoids that are structurally similar to both phyto- and endocannabinoids [1,7]. Two major CBNs, THC and CBD (Figure 1), have been identified as the main bioactive compounds playing critical roles in biological systems and have thus attracted the attention of clinical researchers, owing to their potential as therapeutic agents in the medical and health industries for applications in anti-cancer therapy [7]. For instance, cannabinoids are effective antiemetics against nausea and vomiting in patients with cancer receiving chemotherapy [8,9]. At the same time, several studies have acknowledged the potential anti-cancer properties of cannabinoids against several cancer cell lines [7,10,11]. Reports have also shown evidence of induced apoptosis, reduced cell growth and proliferation, and the anti-inflammatory effects of THC and CBD on human breast cancer and prostate cancer cell lines [12,13,14]. 

In this light, the medical fraternity and related literature has been flooded—all in good spirits—with scientific reports outlining experimental research previewing the anti-cancer capabilities of these cannabinoids (Table 1); scientists and medical professionals have advocated for the adoption of *Cannabis* extracts in medical prescriptions, particularly at the dawn of *Cannabis legalization* in many countries. To date, the US FDA has approved THC-based drugs, such as nabilone and dronabinol, for the treatment of nausea and vomiting induced by chemotherapy, sleep disorders, and the regulation of weight loss in patients with HIV-AIDS [15]. Australia, Canada, and several European nations have greenlit the use of a combined THC-CBD formulation known as nabiximol for the treatment of the spasticity causative of multiple sclerosis, with positive reviews from recent studies [16,17]. However, little is known about the bioactive mechanisms of the actions associated with the activity of these drugs, let alone the sourced *Cannabis* extracts. Patti and colleagues [17] briefly state that “nabiximol’s mechanism of action mimics the effects of endogenous endocannabinoids at cannabinoid CB_1_ and CB_2_ receptors” in the endocannabinoid system. 

These studies, and many more alike, are essential in advancing clinical research and potentially fast-tracking the discovery of new life-saving drugs and the drug discovery process. However, understanding the molecular mechanisms of the actions displayed by these extracts and drug derivatives is essential to exploiting the full medical benefits of these compounds. The current review aims to theoretically elucidate the cellular and molecular mechanisms of CBNs, focusing on CBD and its potential as an anti-cancer and anti-proliferative agent.

## 2. The Endocannabinoid System and Cancer

**Figure 2:**
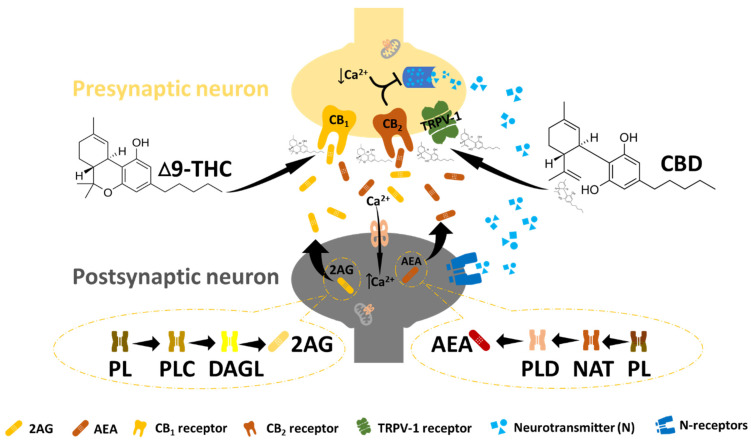
The endocannabinoid system. In ECB signalling, N-arachidonyl ethanolamine (AEA) and 2-arachidonoylglycerol (2-AG) undergo postsynaptic synthesis and are released to bind and activate the CB receptors at the presynaptic site. This leads to a reduction in intracellular calcium at the presynaptic site, followed by the suppression of the neurotransmitter (essential for pain management). Endocannabinoid receptors can also be activated by exogenous cannabinoids such as THC and CBD to promote effects similar to those of endocannabinoids.

The endocannabinoid system (ECS) is a complex cell-signalling system that regulates various physiological and pathological processes in the human body, including mood, appetite, pain, and immune responses (Figure 2) [15]. It comprises cannabinoid receptors (CB_1_ and CB_2_) of the G-coupled protein receptor family, endocannabinoids (eCBNs), and the widely studied endogenous ligands N-arachidonoylethanolamide (anandamide, AEA) and 2-arachidonoylglycerol (2-AG), together with their associated enzymatic machinery (transporters; biosynthetic and degradative enzymes), as well as other enzymes responsible for the regulatory metabolism of eCBNs [15]. The distribution and functional locations of CB_1_ and CB_2_ are surprisingly varied and diverse, with occurrence in the central nervous system (CNS) and the immune system, respectively, making their contributions to regulating various physiological processes interestingly significant [23]. For instance, the activation of the CB_1_ and CB_2_ receptors has been reported to inhibit adenylyl cyclase via the G proteins (Gi/o), leading to the subsequent activation of metabolic pathways, such as the mitogen-activated protein kinase pathway (MAPK), the phosphoinositide 3-kinase pathway (PI3K), and the cyclooxygenase-2 pathway (COX-2) [15,24]. Due to its apparent regulatory involvement in various processes, the ECS has presented itself as a possible therapeutic target through pharmacological modulation, particularly with growing interest in applying cannabinoids to anti-cancer therapy.

However, there are limited data on the mechanism of action of endocannabinoids and the overall role of ECS in cancer, while the available data are seemingly contradictory [25,26]. That is, it is a consensus that the eCBNs and related cannabinoid receptor agonists impact a multitude of cancer-related signalling pathways through the activation of both the CB_1_ and CB_2_ receptors, particularly the pathways associated with anti-proliferative and pro-apoptotic effects [27]. 

On the other hand, Salamat and colleagues [26] have reported on multiple studies that have shown a positive correlation between the ECS and cancer growth and progression. For instance, their observations showed an increased risk of cancer growth and progression due to elevated levels of eCBNs and cannabinoid receptors [26]. The biopsied samples of patients with cancer (glioma, lung cancer, pancreatic cancer, thyroid cancer, mantle cell lymphoma, B-cell non-Hodgkin lymphoma, and human melanoma) showed an increased quantity of CB_1_ and CB_2_ receptors. In contrast, the expression of these receptors was upregulated in breast, pancreatic, and prostate cancer cells [28]. The analysis of human breast and glioma cancer cells revealed the upregulation of TRPV1 (transient receptor potential vanilloid type 1), a noncanonical cannabinoid receptor [29,30]. Additionally, a study by Hart and colleagues [31] demonstrated the oncogenic effects of exogenously applied THC led to the accelerated proliferation of glioblastoma (cell line U373-MG) cancer cells, suggesting the contribution of this CBN to cancer progression in patients instead of apoptosis. However, little evidence supports this claim, except for the concentration-dependent administration of CBNs for cancer treatment. Depending on the concentration, CBNs can inhibit or stimulate cancer cell proliferation [28]. 

So far, the overwhelming evidence of the role of CBNs and the ECS with associated receptors in cancer points to the upregulation of the ECS and the overexpression of receptors as contributing factors towards cancer progression and proliferation. However, given the lack of prior understanding and the elucidation of the homeostatic mechanisms of the ECS, the evidence of the upregulation of ECS-associated cannabinoid receptors in cancer cells thus leaves the impact of the subsequent activation of ECS receptors in the upregulated ECS and cancer up to speculation. What is the relationship between the ECS and cancer; does this refer to the impact of an up- or downregulated ECS on cancer and vice versa, as well as the roles of CBNs in this regard? Deciphering these mechanisms will thus be the focus of this review from henceforth as a by-product of investigating the cellular and molecular mechanisms of CBD’s anti-cancer and anti-proliferative effects.

Contrary to the concerns raised by the studies above, the prospects of CBNs as therapeutic agents have accelerated research into these compounds, with many studies presenting evidence of their anti-cancer properties. Countless studies have reported on the antitumour, anti-proliferation, apoptotic, and overall anti-cancer potential of CBNs. For instance, CBD induces concentration-dependent cell death in breast cancer cell lines [12]. According to the authors, CBD reduces the mitochondrial membrane potential, triggers the translocation of BID to the mitochondria, releases cytochrome c to the cytosol, and, ultimately, activates the intrinsic apoptotic pathway in breast cancer cells. The treatment of human glioma cells with CBD showed the induction of apoptosis [32]; the injection of nude mice with CBD (5 mg/kg) significantly reduced the size of xeno-generated tumours and the number of metastatic nodules in the lungs, displaying the anti-invasive mechanism of CBD [33]. A study by Haustein and co-workers [34] demonstrated the cannabinoid-induced upregulation of ICAM-1 (intercellular adhesion molecule-1) on lung cancer cells to be responsible for increased cancer cell lysis by LAK (lymphokine-activated killer) cells, thus providing evidence of a novel antitumorigenic mechanism and the properties of CBD. 

Several studies have also provided evidence of CBD’s anti-cancer, anti-proliferative, antitumorigenic, antimetastatic, apoptotic, and anti-invasive capabilities in different cancer cell lines, as summarised in Table 1. The recent literature has provided evidence of the therapeutic properties of CBNs in cancer treatments and has promoted the adoption of formulations for clinical trials and applications. However, as previously alluded to, the mechanisms by which CBNs and the ECS with associated receptors impact cancer-related processes are highly complex and rarely elucidated, and our comprehensive understanding of these processes still needs to be achieved [25]. It has also been suggested that these mechanisms are differentially regulated in different types of cancer, impacting various signalling pathways involving multiple CBN receptors [35,36]. As such, the cellular and molecular mechanisms of action of CBNs in cancer, focusing on CBD, will be the focus of this review article from henceforth. CBD is a major bioactive, non-intoxicating CBN and is generally present in higher quantities in *Cannabis* plants than THC is; both traits make this compound very tolerable to patients, and relatively higher doses have less-dramatic side effects, thus providing a favourable safety profile.

## 3. Anti-Cancer and Anti-Proliferation Mechanisms of CBD in Cancer

The mechanisms underlying the antitumor effects of cannabinoids are not yet fully understood, but they may involve multiple pathways. One proposed mechanism is the interaction with the ECS through binding and the subsequent activation of the CB_1_ and CB_2_ receptors. Although they are found in different media and are functional in different parts in the body, the collaborative effects of these receptors essentially culminate in the management of cancer development and proliferation. The CB_1_ and CB_2_ receptors exert anti-inflammatory, pro-apoptotic, and anti-proliferative effects essential in the fight against cancer. CB_1_, found predominantly in the nervous system on the ends of axons, primarily inhibits functional neurotransmitters to reduce pain in patients [37]. On the other hand, the CB_2_ receptors are active in immune cells, where they regulate cytokinesis, ultimately reducing the frequency of cell division to affect the anti-proliferation of cancer cells via an apoptotic signalling mechanism [37,38]. 

**Figure 3:**
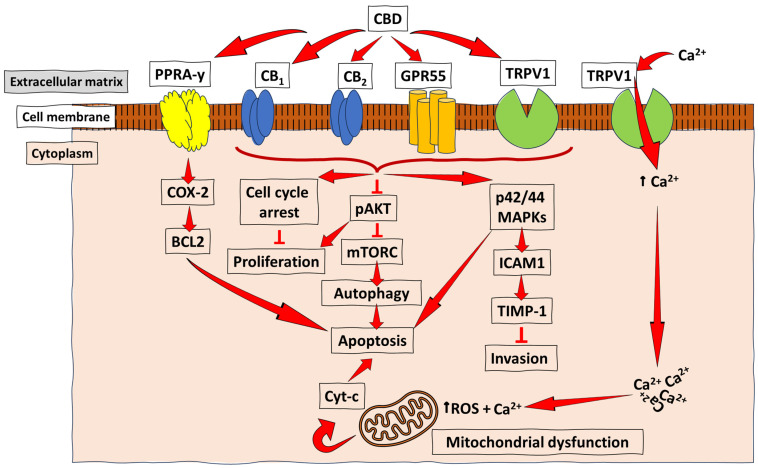
Schematic representation of the anti-cancer mechanisms of CBD. CBD acts as an agonist (activator) for the receptors TRPV1 and PPARγ in most cancers, and with a low, but effective, affinity for the CB receptors (CB_1_ and CB_2_) to promote the downstream activation of pathways essential for cancer cell autophagy, reduced proliferation, and invasion, as well as apoptosis. At the same time, acting as an antagonist for GPR55 leads to inhibiting tumour cell growth. The PPARγ-mediated COX-2-BCL2 pathway has been reported mainly in lung cancer. At the same time, CB receptor- and TRPV1-mediated CBD signalling were demonstrated in breast cancer, prostate cancer, and gliomas, among other types of cancer, which lead to apoptosis/reduced invasion/reduced proliferation and autophagy through varying pathways, such as the ICAM-1, the pAKT inhibition of mTORC, and the induction of cell cycle arrest. ↑ = increase/upregulation; ┴ = inhibition.

Even so, it has been revealed that CBD has a low affinity for CB_1_ and CB_2_ receptors. Instead, for optimal functionality, the CBN interacts with other receptors, such as G protein-coupled receptor 55 (GPR55), transient receptor potential channels (TRPV1), and peroxisome proliferator-activated receptors (PPARs) [15,24]. These interactions can modulate various signalling pathways involved in cancer. Molecular studies have further shown that CBD affects multiple signalling pathways in cancer progression and cell proliferation, including the PI3K/AKT/mTOR pathway, the MAPK/ERK pathway, and the NF-κB pathway (Figure 3). CBD’s modulation of these pathways can lead to the inhibition of cell growth, the induction of apoptosis (programmed cell death), and the suppression of tumour angiogenesis (the formation of new blood vessels to support tumour growth) [39]. 

A recent study by Go and colleagues [39] showed that a treatment with CBD demonstrated apoptotic and autophagy events that suppressed the growth and proliferation of head and neck squamous cell carcinomas by promoting the activity of *DUSP1* (dual-specificity phosphatase 1), which is capable of inactivating the MAPK isoforms in oncogenic signalling. Zhang and co-workers [40] observed the anti-proliferative effects of CBD through the upregulation of the ataxia telangiectasia mutated (ATM) gene and the expression of the p21 protein and the downregulation of the p53 protein, leading to a decrease in CDK2 and CCNE, with subsequent G_0_/G_1_ phase cell cycle arrest [40]. Another proposed mechanism is the inhibition of angiogenesis (the formation of new blood vessels), which is essential for tumour growth and metastasis, as described by Massi and colleagues [23], Śledziński and colleagues [15], Heider and colleagues [37], and Hinz and Ramer [4]. Briefly, the in vitro and in vivo anti-angiogenic properties of CBD were associated with the receptor-mediated down-modulation of several angiogenesis-associated molecules, such as MMP2 and MMP9, endothelin-1 (ET-1), platelet-derived growth factor-AA (PDGF-AA), as well as the inhibition of the hypoxia-inducible transcription factor HIF-1α, one of the most critical stimuli for cell survival, motility, and tumour angiogenesis [4,23,41]. Some studies over the years have discovered the differential regulation of molecular pathways and cellular mechanisms of CBD in different cancers, suggesting a cancer-specific mode of action, which is a reminder that cancer is a heterogeneous disease. Henceforth, this review will evaluate CBD’s efficacy and detailed mechanisms of action in common human cancer lines.

### 3.1. Breast Cancer

The inhibitory effects of CBD have primarily been studied using breast cancer models, following some comprehensive reviews of the impact and mechanisms of CBD in breast cancer by Almeida and co-workers [42]. The authors present four subtypes of breast cancer, luminal A, luminal B, human epidermal growth factor receptor 2 positive (HER2^+^), or triple negative (TNBC), of which luminal tumours are the most prevalent, accounting for 60–73% of all recorded cases, with luminal B breast cancer as the most aggressive. Therefore, CBD’s proposed mechanisms of action are expected to differ slightly among the various cancer types, yet all may involve the differential regulation of the ECS. It has been well established that the ECS is altered in cancer cell lines closely associated with tumour aggressiveness due to elevated concentrations of CBNs and the CB receptors [15]. In breast cancer cases, a correlation has been established between CB2 and tumour aggressiveness as compared to CB_1_, and due to the low affinity between CBD and the CB receptors, the involvement of TRPV1 receptors has been suggested to play a crucial role in CBD’s anti-cancer activity on breast cancer cells.

The earlier studies on TNBC showed that the anti-proliferative effects of CBD occurred in a biphasic manner through the direct activation of the TRPV1 receptor and peroxisome proliferator-activated receptors (PPARs) in MDA-MB-231 cells [43,44]. The activation of TRPV1 (a member of the mammalian TRP family of ion channels) leads to an influx of calcium ions (Ca^2+^) into the cell and mitochondrial Ca^2+^ overload, leading to mitochondrial damage [the disassembly of mitochondria–dynamin-like GTPase (Opa1) oligomers] and the subsequent release of cytochrome *c* through the now-oligomerized pro-apoptotic factors, such as the BAX and BAK mitochondrial pore regulators, which is followed by the activation of caspases 8 and 3 to initiate the intrinsic apoptosis pathway [1,45]. Moreover, cytochrome *c* further binds to IP_3_Rs (inositol 1,4,5-trisphosphate receptors), thus blocking the Ca^2+^-dependent inhibition of channel function, prolonging the cytosolic Ca^2+^ influx and mitochondrial Ca^2+^ overload, and amplifying apoptotic signalling [45,46]. 

CBD was also found to facilitate a PPAR-mediated apoptotic pathway in MDA-MB-231 cells and ER^+^ T-47D cells; the study by Sultan and co-workers [47] demonstrated the upregulation of the transcription factor PPAR_y_ in breast cancer cell lines accompanied by the downregulation of mTOR and cyclin D1. The anti-proliferative effect from the activation of PPAR-γ is fulfilled by the subsequent ablation of cyclin D1 (a family of positive regulators for cell progression) and the blocking of the mTOR signalling pathway, which leads to the downregulated proliferation, migration, and invasion of breast cancer cells, as well as the upregulation of apoptosis and autophagy [48]. On the other hand, the combined induction of endoplasmic reticulum stress (ER stress) and the inhibition of the mTOR-associated AKT signalling pathway has also been reported to induce autophagy and apoptosis in studies by Shrivastava and colleagues [12], Murase et al. [49], and Elbaz et al. [50], while reducing cancer cell proliferation and cell survival through the inhibition of the NF-kB signalling pathway. 

These effects were reported as a result of the CBD inhibition of EGF-R (epidermal growth factor receptor) activation, which is responsible for the growth and metastasis of tumours [37]. Similar findings were reported by Milian and co-workers [51] in an in vitro study of lung cancer (further discussed in Section 3.2). The inhibition of EGF-R also reduces breast cancer cell metastasis, invasion, and migration by inhibiting the RAF1-MEK/ERK signalling pathway, which promotes cancer cell proliferation and migration capabilities [52]. Similar signalling pathways and the mode of action of CBD as an anti-proliferative and anti-cancer agent have been reported in luminal and HERS2^+^ tumour cells [53,54,55]. 

### 3.2. Lung Cancer

According to Siegal and co-workers [56], and as further highlighted by Heider et al. [37], lung cancer cells exist in two distinct forms differentiated by the rates of growth and metastasis, as in non-small-cell lung cancer (NSCLC), making up the majority of cancer cells (~83%) and the faster growing and metastatic small-cell lung cancer (SCLC; ~13%). The NSCLC cell lines have, so far, been the target cell lines for studies on CBD-based anti-cancer efficacy and mechanisms of action. Ramer and colleagues [33,57,58] showed that treating NSCLC cell lines (A549 and H460) with CBD reduced the cell viability in both the cell lines. The authors revealed that the efficacy of CBD also increased with the concentration administered. Comparatively, 29% and 63% reductions were observed at 0.001 µM and 0.1 µM CBD, respectively. The studies also showed the expression of CBD receptors CB_1_, CB_2_, and TRPV1 from the lung cancer cell lines as an entry point for CBD [33]. A literature survey has revealed three significant modes of action; in lung cancer, CBD induces apoptosis via cyclooxygenase 2 (COX2) and PPARɣ signalling, reduces metastasis by upregulating the expression of surface protein intercellular adhesion molecule (ICAM-1), and increases the susceptibility of lung cancer cells to adhere to and subsequently be lysed by lymphokine-activated killer (LAK) cells [2,15,59]. 

The detailed mechanism of CBD-induced cyclooxygenase 2 (COX-2) and PPARɣ signalling-mediated apoptosis in lung cancer has not been extensively elucidated. The documented mechanisms have, thus far, been based on pre-clinical trials and hypothetical frameworks, while only a few studies can be found. A study by Ramer and co-workers [33] showed that CBD-induced apoptosis is associated with the upregulation of the pro-apoptotic markers, COX-2 and PPARɣ. These proposed novel mechanisms were revealed from the treatment of A549 and H460 NSCLC lines and primary lung tumour cells with CBD, which induced the upregulation of COX-2, followed by elevations of COX-2-derived PGE_2_, PGD_2_, and 15dPGJ_2_, leading to apoptotic cell death. These findings were evidenced by the observed attenuation of CBD’s pro-apoptotic and cytotoxic effects when the COX-2 and PPARɣ activities were suppressed with active antagonists or siRNA in NSCLC lines [1,33]. The mechanistic nature of the pro-apoptotic COX-2 and PPARɣ signalling pathways follows the activation of the COX-2 enzyme by CBD, which leads to the production of COX-2-associated prostaglandins (PGs) (PGE_2_, PGD_2_, and 15dPGJ_2_), which are also considered to be PPARɣ ligands [60]. 

The accumulation of PGs activates the transcriptional activity of PPARɣ; this is followed by the formation of the death-inducing signalling complex (DISC) consisting of the death adaptor protein FADD, which recruits and cleaves the pro-caspase-8 to produce caspase-8, an apoptosis initiator protein that can further activate the downstream caspases (-3, -6, and -7) to activate the extrinsic apoptotic pathway [60]. On the other hand, activated caspase-8 facilitates the cleavage of Bid (BH3 interacting domain) to a truncated (tBid), a pro-apoptotic member of the Bcl-2 protein family which promotes the activation and insertion of Bax into the mitochondrial membrane, leading to mitochondrial damage and subsequent cytochrome *C* release to activate the apotome (pro-caspase-9 and Apaf-1 complex) through the intrinsic apoptotic pathway. Activated caspase-9 further activates the downstream caspases (-3, -6, and -7) to induce apoptosis [18,60]. 

CBD has been shown to exhibit anti-invasive and antimetastatic properties against lung cancer cells in a dual mechanism involving intercellular adhesion molecule-1 (ICAM-1). According to Ramer and colleagues [33], and as further reviewed by Heider et al. [37], CBD significantly attenuates ICAM-1-dependent lung cancer (A549, H358, and H460 NSCLC) cell invasion via cannabinoid receptors, TRPV1, and p42/44 MAPK. At an increased concentration, CBD has been shown to significantly decrease the size of xenografted tumours and metastatic nodules through ICAM-1 and TIMP-1 (tissue inhibitor of metalloproteinases 1) as the key molecular targets of the anti-invasive mechanism of CBD [33,37], preventing cancer cell adhesion to non-cancer cells and reducing cell invasion and metastasis. 

Interestingly, CBD can also promote cancer cell adhesion as much as it does prevent the process in other instances. Haustein and colleagues [34] reported that CBD promotes the expression of ICAM-1 on lung cancer cells as a target for their anti-invasive and antimetastatic actions. Here, the CBD treatment of lung cancer cell lines (A549 and H460) enhanced the adhesion of such cell lines to lymphokine-activated killer (LAK) cells, resulting in LAK cell-mediated cytotoxicity (apoptosis) and the lysis of cancer cells and the subsequent attenuation of lung cancer cell invasion and metastasis [34]. These findings were further confirmed by the profound decrease and complete reversal of any cancer cell lysis by the LAK cells when the expression of ICAM-1 was blocked by transfecting the cells with ICAM1 siRNA. The study further reported the cancer-specific CBD-mediated LAK cell adhesion and lysis of cancer cells, without any indication of the LAK cell-mediated lysis and upregulation of ICAM-1 of the non-tumour bronchial epithelial cells.

### 3.3. Prostate Cancer

Several studies have identified several CBN-receptor-regulated pathways that directly regulate cell proliferation and cell death in prostate cancer. In addition to the inhibition of the adenylyl cyclase resulting in reduced the cAMP levels, which can lead to cell death, the activation of CBN receptors can also mitigate cell proliferation and enhance cell death through the regulation of the extracellular-signal-regulated kinase (ERK) associated with mitogen-activated protein kinase (MAPK) and p38 MAPK, phosphatidylinositol 3-kinase (PI3K)/Akt, focal adhesion kinase, and the ceramide and reactive oxygen species (ROS) pathways [61,62,63]. Some of these pathways are further discussed below. 

The effects of CBNs on peripheral tissue were first studied by Ruiz and colleagues [64]. Following reports that CBNs such as anandamide are produced in the testes and that CB_1_ receptors have been found expressed in reproductive organs, the authors explored the effects of CBNs on prostate cells. The study was performed on a PC-3 human epithelial cell line derived from prostatic metastasis, on which a treatment with THC was observed to induce apoptosis through a receptor-independent pathway, which represents the only revelation thus far [64]. A later study from the same colleagues further shed light on the potential mechanisms of eCBNs in anti-prostate cancer activity; here, the authors revealed that the activation of CBN receptors with the eCBN 2-Arachidonoylglycerol (2-AG) in PC-3 cell lines promotes the PI3K/Akt pathway and the subsequent activation of the Raf-1/ERK1/2 signalling pathway [65], which represents the first look at the molecular mechanism of these activities. 

Many earlier studies used agonists to activate the CBN receptors to unravel the mechanism of ECS regulation in prostate cancer. For instance, Safraz and co-workers [66] observed that the CB receptor agonist induces apoptosis in human prostate cancer cells (LNCaP) via ERK1/2-mediated cell cycle arrest. The authors treated LANCaP cells with WIN-55-212-2 (a mixed CB_1_ and CB_2_ agonist) as a follow-up to their previous study on using a cannabinoid receptor as a novel target for the treatment of prostate cancer [66], reporting several significant events that resulted in the inhibition of cell growth and the induction of apoptosis; these activities included “(i) an arrest of the cells in the G_0_/G_1_ phase of the cell cycle; (ii) an induction of p53 and p27/KIP1; (iii) downregulation of cyclins D1, D2, E; (iii) decrease in the expression of cdk-2, -4, and -6; (iv) decrease in protein expression of pRb; (v) downregulation of E2F (1–4); and (vi) decrease in the protein expression of DP1 and DP2”. Additionally, the sustained inhibition of PI3/Akt and the activation of the ERK1/2 pathways were observed, as was previously reported by Nithipatokom and co-workers [65], which leads to cell cycle dysregulation and arrest at the G_0_/G_1_ phase, ultimately resulting in the induction of apoptosis. The evidence strongly indicates that focusing on the CBN receptors could be an effective remedy for prostate cancer. This prompts us to consider whether CBNs can produce effects comparable to those of other agonists. Furthermore, the studies have illuminated the potential mechanisms by which CBNs can function as anti-cancer agents.

Though they are very limited, recent studies have thus explored the effects of CBNs on prostate cancer, providing further evidence of their efficacy in reducing cancer progression. A 2022 study by Motadi and Moleya [20] examined the antitumor activity of CBD in the PC-3 cells of mouse models; the results showed the suppression of the Bcl2 (Bak- and Bax-mediated) signalling pathway coupled with an increase in caspase3/7 activity and the upregulation of p53 and Bax mRNA expression. This groundbreaking study demonstrated CBD as a viable therapy for prostate cancer, citing the observed reduction in the growth of tumours in the mouse models following treatment with the CBN, which could form the basis for future exploration. The scarcity of CBD-focused research on prostate cancer is thus a cause for the extrapolation of facts based on studies performed on CBN-receptor-dependant agonists; that is, the collective evidence of the anti-cancer and pro-apoptotic efficacy of CB receptor agonists, such as the synthetic CBN (WIN 55-212-2), greatly highlight the potential anti-cancer characteristic of CBD, as per the pathways previously discussed. To illustrate, Singh and colleagues [67] showed the oxidative stress signalling pathway as a channel for CBN-induced apoptosis in prostate cancer rather than only as a proposed mechanism derived from related cancer(s) studies. Here, the CB receptor-mediated perception of CBNs induces the accumulation of reactive oxygen species (ROS) that cause a reduction in the mitochondrial membrane potential (mV) of prostate cancer cells, which releases cytochrome c and the subsequent activation of caspase9 and caspase3 to promote apoptosis. As such, further in-depth studies are still required to fully elucidate the mechanisms of action of CBD for prostate cancer treatment.

### 3.4. Glioma

CBNs have been significantly studied in gliomas due to their previously reported medical capabilities and the urgent demand for unmet medical needs as glioma resists the current anti-cancer therapies [68]. The seriousness of the cancer can justify the extent to which medical scientists have explored CBNs for glioma treatment; it is the most common (and most aggressive) primary brain malignancy. With poor rates of prognosis and only a 4–5% survival rate within five years [1], medical research must make great strides towards a viable and sustainable resolution. Several studies have reported (in vitro and in vivo) the anti-invasive, anti-cell migration, anti-proliferative, and pro-apoptotic actions of CBD in glioma cancer cells [69,70,71,72]. Some of these studies have also shown CBD to decrease the tumour’s size, vascularization, growth, and weight, while promoting tumour regression. Contrary to the assumptions of CB (1 and 2) receptor-dependant mechanisms, Torres and colleagues [69] revealed the TRPV2-dependant anti-proliferative pro-apoptotic actions of CBD on glioma, serving as the initial site of investigation for several of the proposed cellular and molecular mechanisms. 

A study by Ivanov and co-workers [32] showed that CBD, in a similar fashion to glioma cancer treatments such as ɣ-irradiation, leads to the substantial upregulation of TNF/TNFR1 and TRAIL/TRAIL-R2 signalling along with the death receptor (DR5) within the extrinsic apoptotic pathway as one of multiple cell death-inducing signalling mechanisms of CBD in human glioblastoma. The second aspect involved the significant upregulation of the active (phosphorylated) JNK1/2 and MAPK p38 levels and the resultant downregulation of the active phosphor-ERK1/2 and phosphor-AKT1 levels, as has previously been reported by Carracedo et al. [73] and Vara et al. [74]. MAPK p38 and JNK were found to regulate the endogenous TRAIL expression mentioned above, thus establishing a possible synergy between the two pathways. In a follow-up study from the same group, Ivanov et al. [75] further revealed that the CBN also induced the activation of the JNK-AP1 and NF-κB pathways in glial cells, leading to apoptosis. The results showed both apoptotic and non-apoptotic inflammation-linked cell death due to the upregulation of the percentage of G2/M-arrested cells, the blockade of cell proliferation, and the massive production of pro-inflammatory cytokines.

Additional mechanisms of action have been brought to light, including inhibiting the critical regulatory pathways in cancer cells. For instance, CBD was reported to inhibit the PI3K/AKT signalling pathway, a major signalling pathway in various cancer cells responsible for hallmark activities, such as cell survival, metastasis, and metabolism [76]. According to Hennessy et al. [77], PI3K is essential for the activation of phosphatidylinositol-4,5-bisphosphate (PIP2) to generate phosphatidylinositol-3,4,5-trisphosphate (PIP3), which then recruits and activates Akt, among other oncogenic signalling proteins. Activated Akt then affects the downstream proteins such as mTOR to promote cancer progression [76,77,78]. This claim is supported by evidence suggesting that PI3K/AKT signalling can stimulate gliomagenesis [79]. Therefore, the cascading inhibition of the PI3K/Akt signalling pathway can be an effective treatment strategy for cancer. Nabissi et al. [80] evaluated the role of CBD and the PI3K/AKT pathways in inhibiting glioma stem-like cell (GSC) proliferation. The study showed that the TRPV2-mediated, PI3K/Akt-dependent perception of CBD by GSC lines induced the expression of acute myeloid leukemia-1A (Aml-1a), a class of transcription factors that regulates the differentiation of hematopoietic stem cells, thereby inducing autophagy. 

One of the critical downstream targets of the PI3K/Akt pathway is the mammalian target of the rapamycin (mTOR) signalling pathway, which positively regulates cell growth (through the tight rotation of the lipid metabolism pathway, which is an essential process for cancer growth) and proliferation by promoting protein synthesis and the inhibition of autophagy [81]. This presents mTOR as a critical target for anti-cancer strategies. In light of the evidence suggesting the involvement of the PI3K/AKT/mTOR pathway in GBM, Eckerdt and colleagues [82] evaluated the impact of dual PI3K/mTOR pathway inhibition in GBM and GSCs, in which simultaneous mTOR inhibition was found to potentiate the antineoplastic effects of PI3Kα inhibition. The study further focused on PI3Kα, a subtype of the PI3K kinases, which GBM and the GSCs depend on for increased signalling. The authors thus propose the alpha catalytic PI3K isoform as a unique therapeutic target in proneural GBM. Nabissi et al. [80] also showed that inhibiting the mTOR regulatory pathway is essential in the CBD-mediated autophagy of the GSCs. The process begins with CBD-dependent TRPV2 activation that initiates the recruitment of the eIF4F complex (the cap-binding protein eIF4E, the scaffolding protein eIF4G, and the ATP-dependent RNA helicase eIF4A), which potentiates the translation of mRNAs that affect stress-mediated differentiation, cellular growth, apoptosis, and the autophagy of the GSCs.

**Figure 4:**
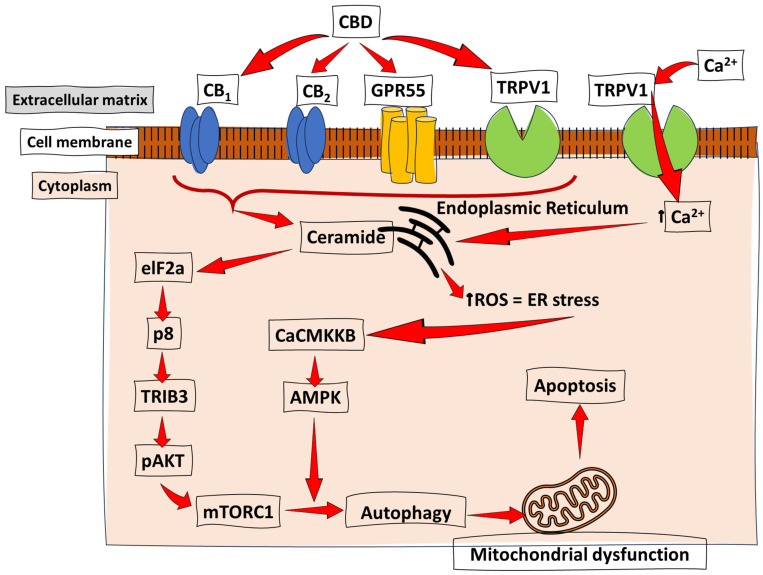
Cannabinoid-induced apoptosis through ER stress and autophagy. Studies on several cancer types have revealed CBD’s proposed mechanism of action in the induction of apoptosis. The CB receptor activation promotes apoptotic events, which induces ER stress and activates calcium/calmodulin-dependent protein kinase kinase-β (CaCMKKβ) and AMP-activated protein kinase (AMPK). This pathway is accompanied by the previously discussed AKT-mediated inhibition of the mTORC pathway to induce autophagy, mitochondrial dysfunction, and, ultimately, apoptosis. ↑ = increase/upregulation.

Moreover, an interrupted Akt/mTOR pathway can induce the autophagy of tumour cells by stimulating ER (endoplasmic reticulum) stress in human glioma cells. This action was reported by Salazar et al. [19] when the molecular basis underlying CBN-induced autophagy was investigated (Figure 4). The study showed that the activation of the CB receptor stimulates ceramide accumulation and the phosphorylation of the eukaryotic translation initiation factor 2α (eIF2α), thereby activating ER stress through the upregulation of the stress protein p8 and its ER stress-related downstream targets ATF4, CHOP, and tribbles homolog 3 (TRB3). Increased TRB3, through its subsequent interaction with Akt, thus facilitates the inhibition of the Akt/mTOR pathway via the decreased phosphorylation of Akt kinase, leading to induced autophagy and apoptosis. In another study by Solinas et al. [83], the ability of CBD to affect (interfere with the expression of proteins involved in tumour growth and spreading) the pro-tumoral PI3K/Akt pathway in two different glioma cell lines (U87-MG and T98G cells) was investigated. CBD was found to strongly downregulate the PI3K/Akt pathway alongside the ERK pathway, with a significant reduction in tumour cell proliferation, and hampered the capabilities of tumour cells to evade cell cycle arrest and apoptosis. Hence, the CBD-pharmacological inhibition of the PI3K/Akt signalling pathway represents a viable case for its potential therapeutic use to treat gliomas.

## 4. Overview of Anti-Cancer Capabilities of CBD in Other Cancers

The current review not only provides evidence of the efficacy of CBD as an anti-cancer agent in several cancer cell lines, some of which have been discussed in detail, but also highlights some of the major cellular and molecular mechanisms involved. These mechanisms have been largely elusive, and thus very well elucidated. However, revelations into the modes of action of CBD in cancer must be made for optimized targeted drug discovery and development processes and the design of new therapeutic strategies for cancer treatment or combination therapies. Such insights may direct research into the benefits, such as optimal efficacy, dosages, and safety assurance, particularly in clinical trials. Pre-clinical trials have demonstrated the potential of CBD as an inhibitor of tumour growth, angiogenesis, invasion, and proliferation and as a promoter of autophagy and apoptosis. 

Various cellular and molecular strategies employed by CBD in different cancers have also been unravelled by several studies, as reviewed above; simultaneously, it has become evident that CBNs activate and promote similar pro-apoptotic mechanisms in various cancer cells. For instance, the CBD-induced pro-apoptotic mechanisms in glioma and pancreatic cancer are both a result of the CB_1_ and CB_2_ perception of the compound, which is followed by ceramide synthesis, induced ER stress, and the upregulation of p8–TRIB3, which leads to Akt inhibition and subsequent autophagy to enable apoptosis [19,73]. A similar signalling cascade was reported in hepatocellular carcinoma by Vara et al. [74], wherein CBD-induced ER stress activates calcium/calmodulin-dependent protein kinase kinase-β (CaCMKKβ/CAMKK2) and AMP-activated protein kinase (AMPK), which when coupled with p8–TRIB3, leads to autophagy and apoptosis (Figure 4) [68,84]. 

Likewise, studies on leukaemia revealed CBD-mediated apoptotic modes of action similar to the mechanisms proposed for prostate cancer. It was shown that the CBN treatment of the human leukaemia cell line Jurkat expressing CB_2_ receptors leads to receptor-dependent ceramide biosynthesis and the downregulation of the Raf-1/mitogen-activated protein kinase/ERK kinase (MEK)/ERK/RSK pathway to induce apoptosis [21]. Similarly, Herrera et al. [85] showed that CBN exerts CB-dependent pro-apoptotic actions in the tumour cells of the human leukaemia cell line Jurka through the ceramide-mediated stimulation of the intrinsic mitochondrial pathway. ER, stress, p8–TRIB3 induction, and autophagy are yet to be fully elucidated in prostate and leukaemia cancer cell lines. 

The anticarcinogenic effects of CBD have recently been reported in different types of skin cancers at varying levels of cancer progression; however, the information on these possibilities is limited, and thus is yet to be thoroughly understood. According to a recent study, cannabinoids have shown a considerable impact as a potential treatment and prophylaxis of tumours associated with skin diseases, inclusive of the inhibition of tumour growth, proliferation, invasion, and angiogenesis, as well as inducing apoptosis and autophagy [86]. Skin cancers are categorised into three: cutaneous melanomas, basal cell carcinomas, and squamous cell carcinomas; the latter two are termed non-melanoma skin cancers and typically recorded with a meagre mortality rate [86,87]. In contrast, melanoma (the most aggressive form of skin cancer) continues to rise in global incidence and mortality rates, accounting for 80% of skin cancer-related deaths [88,89], prompting the dedication of resources to melanoma-associated research, particularly with cannabinoids (CBD and THC) as potential therapeutic agents, following evidence of significant adverse side effects and only moderate progress in terms of patient survival after using classical chemotherapeutic agents [86,90]. 

Several studies have shown the regulation of the ECS cannabinoid receptors in skin cancer tissues, where such elements as the CB receptors were upregulated in the melanoma tissue [91]. The melanoma cancer cell lines (B16 and A375) express the endocannabinoid receptors CB_1_ and CB_2_ [1,86], the CBD-dependent activation of which has decreased melanoma growth proliferation, angiogenesis, and metastasis in vivo. A study by Blázquez and colleagues [92] investigated CBN receptors as novel targets for the treatment of melanoma. The activation of the CB_2_ receptor was found to lead to decreased growth, proliferation, angiogenesis, metastasis, and the increased apoptosis of melanomas in mice in a similar fashion as seen in breast cancer cell lines [92]. This action was observed through the p8–TRIB3-induced inhibition of the pro-survival Akt signalling pathway, resulting in autophagy and, ultimately, apoptosis [23,68,92]. 

An investigative study examined the impact of CBD on the growth and spread of malignant melanoma cells in a mouse model using B16F10 mouse melanoma cells injected into C57BL/6 mice. The results indicated a noteworthy reduction in tumour size among the mice treated with CBD compared to those in the control group [93]. Additionally, the CBD-treated animals exhibited a significantly prolonged survival time compared to that of the control group, although the comparison group treated with cisplatin showed the longest survival time. Conversely, the CBD-treated mice show an improved quality of life and physical performance [93]. Furthermore, a study by Burch and co-workers [22] revealed that CBD oil effectively hindered the growth of B16 melanoma cells in vitro. However, the precise proapoptotic mechanism of CBD in melanoma cells remains unknown, yet efforts to elucidate these mechanisms have not ceased. A recent and detailed in vitro study on the effects of CBD in melanoma cells showed the significant inhibition of melanoma cell growth, reduced cell viability, invasion, metastasis, and the induction of apoptosis through the ER stress and MEK signalling pathways [22]. It has been confirmed that the CBD-stimulated inhibition of Akt activates the cyclin-dependent kinase inhibitory proteins p21 and p27, causing the phosphorylation of the retinoblastoma protein, and eventually cell cycle arrest and apoptosis [15,94]. Most recently, Richtig and colleagues [95] reported a CBD and CBD-THC-mediated reduction in cell viability from different melanoma cell lines by activating the CB1, TRPV1, and PPARα receptors and activating cytochrome c-mediated cell death. A similar mechanism has been reported in colon (colorectal) cancer [CRC], where the downregulation of the PI3K/AKT pathway and the upregulation of Caspase-3 was observed following CRC cell treatment with CBD [96]. At the same time, a different study showed that CBD induces apoptosis in an ROS-dependent manner, resulting from mitochondrial damage, ER stress followed by cytochrome c release, and subsequent caspase-9 activation [97], similar to the mechanisms observed in other cancers above.

Overall, the quest to elucidate CBD’s cellular and molecular mechanisms as an anti-cancer agent has revealed insightful information into the modes of action of the compound. Several cellular targets have been identified, including CB_1_ and CB_2_, though with a relatively low affinity to some cancer cells other than lung, colorectal, and prostate; however, other targets, such as TRPV1, TRPV2, and GPR55, which are highly expressed in cancer cells have been identified as alternatives. The cellular response to CBD treatment is quite complex, with varying mechanisms in different cancers. However, the surveyed literature presents a near-standard mode of action that sees triggered ROS production and induced ER stress, both from the disruption of intracellular calcium homeostasis and mitochondrial damage. The upstream regulation of ER stress and ROS remains largely elusive, mainly due to the diverse CBD mechanisms of action and the dependence/independence on various receptors, as revealed above.

## 5. CBD and THC: Why One, Not the Other, or Both?

A deeper look into the intricacies of CBD vs. THC is beyond the scope of the current review, particularly regarding their specified cellular and molecular mechanisms of action, pharmacological properties, and potential therapeutic applications. Nonetheless, the scientific literature has favoured CBD over THC for several important and objective reasons. Both CBD and THC exert their anti-cancer effects through intricate cellular and molecular mechanisms, although their approaches diverge, with significant implications. This article outlines that CBD’s anti-cancer activity encompasses multifaceted pathways, including apoptosis induction, cancer cell proliferation inhibition, and angiogenesis and metastasis suppression. CBD induces apoptosis in cancer cells by modulating the expression of pro-apoptotic and anti-apoptotic proteins, activating both the intrinsic mitochondrial and extrinsic death receptor pathways [37,98,99]. Furthermore, CBD disrupts cancer cell proliferation by regulating cell cycle progression and interfering with the signalling pathways crucial for cell growth and survival, such as the PI3K/Akt/mTOR pathway. Additionally, CBD exhibits anti-angiogenic properties by inhibiting the expression of pro-angiogenic factors and impeding the formation of new blood vessels, thereby hindering tumour progression and metastasis [37,99].

In contrast, THC primarily engages the cannabinoid receptors, notably CB_1_ and CB_2_, to exert its anti-cancer effects. The activation of these receptors triggers signalling cascades, leading to apoptosis induction, cell proliferation inhibition, and cell migration and invasion modulation. Through CB_1_ and CB_2_ receptor activation, THC induces apoptosis in cancer cells and impedes their proliferation by interfering with the key signalling pathways involved in cell growth and survival, such as the MAPK/ERK and PI3K/Akt pathways. Moreover, THC influences the tumour microenvironment by modulating the immune responses and cytokine production, thereby contributing to its anti-cancer efficacy [37,99]. 

While both the compounds share the overarching goal of combating cancer, CBD and THC diverge in their mechanisms of action. CBD exhibits a multi-targeted approach, targeting various pathways involved in cancer progression. In contrast, THC primarily relies on cannabinoid receptor (CB_1_ and CB_2_) activation to exert its effects, which is further associated with undesirable psychotropic effects (mediated by CB_1_) characteristic of marijuana. Some reports have also shown THC to exhibit the impact of cognitive dysfunction, particularly the loss of short-term memory consolidation [100]. This contrast extends to their cytotoxicity profiles, with CBD presenting a safer option due to the minimal psychoactive effects compared to those of THC. Nonetheless, the pre-clinical studies hint at potential synergistic effects when CBD and THC are combined, suggesting that their distinct mechanisms may complement each other to enhance anti-cancer efficacy, without exacerbating the psychoactive side effects of one. For instance, CBD was shown to reduce unpleasant THC-induced effects, such as psychological reactions, anxiety, tachycardia, and hunger [100,101], through the CBD-THC competitive binding affinity to the CB_1_ receptor. Additionally, a THC-CBD combination was an efficient treatment for pain relief in patients with lung cancer compared to THC treatment alone [51]. A dosage test also showed a combined THC-CBD treatment (10µM each) was the most effective at inhibiting NSCLC cell proliferation compared to separate treatments of the CBNs at 30µM each and in downregulating the gene expression of epidermal growth factor receptor (EGFR), which was not observed in the treatments with cannabinoids alone. The synergistic activities of CBD and THC highlight promising prospects for combination therapy; therefore, understanding the intricate cellular and molecular mechanisms underlying the actions of CBD and THC is crucial for optimizing their therapeutic potential in cancer treatment.

## 6. Concluding Summary 

The body of literature reviewed above presents an overarching theme, highlighting the theoretical framework on the mechanistic nature of CBD as a potential anti-cancer agent. Groundbreaking research has shown that phytocannabinoids demonstrate robust anti-cancer capabilities, mainly through anti-proliferative, anti-invasive, and proapoptotic mechanisms in different cancer types. CBD employs a variety of anti-cancer mechanisms in other cell lines (receptor-dependent/-independent and pathway specificity), suggesting that the modes of action of this compound are cancer-specific. Additionally, this compound has been shown to have multiple targets. However, a closer examination of these mechanisms indicates cell cycle arrest, autophagy, cell death, or a combination of these activities as the end product in all the cancer cells that have been investigated. Therefore, the varying mechanisms employed in different cell lines, i.e., the molecular pathways associated with the anti-cancer activities of CBD, effectively induce apoptosis, which suggests a converging focal point (induced ER stress and autophagy) that can be investigated further as a potential primary target for anti-cancer therapy.

Moreover, this compound’s modes of action suggest capabilities in modulating multiple pathways that control tumorigenesis, effectively displaying versatility in its mechanisms of action. The targeted nature of CBD’s mode of action has been of interest, particularly with the progressive move from non-specific chemotherapies to molecularly targeted inhibitors. Due to its targeted nature, CBD has milder effects on normal cells from the same tissue/organ compared to those of cancer cell lines. On the other hand, the role of CBD and its associated receptors in the modulation of cancer cell activity has been controversial, with clear and distinct roles in some cancers (lung, leukaemia, and colon), while showing some murky and inconsistent actions. This observation calls for more specialized and targeted research on the activity of CBD in certain cancers in association with the relevant receptors, which would further provide insights into the detailed mechanisms involved, essential for the strategic design and development of clinical trials. 

Additionally, the clinical applications of CBD are considerable, given its low toxicity to dosage and efficacy ratios due to the non-toxic characteristics of this compound. Moreover, strategies for multi-target therapies provide an opportunity for combined CBD treatments with other medicines, wherein co-administration can considerably offer an effective response that is the sum of the individual therapies. Overall, the studies presented herein have given insights into the potential of CBD as an anti-cancer agent and a possible sustainable alternative to current treatments. 

## Figures and Tables

**Table 1:** Effects of cannabinoids (particular focus on CBD) in various types of cancer. This table shows evidence of CBD inducing similar anti-cancer and pro-apoptotic mechanisms for different kinds of cancer.

Cancer	Receptor	Molecular Cell Signalling	In Vitro/Vivo Effect on Cancer Cells	Autophagy Apoptosis	Reference
Cervical	ND	↑ p53, caspase3/7 and bax ↓ ATP levels	↓ Proliferation	Apoptosis	[11]
Breast	ND	↓ RBBP6 expression ↑ p53 gene expression	↓ Cell growth ↓ Cellular proliferation	Apoptosis	[10]
Lung	ND	↑ caspase-9/-3 ↑ caspase-8/Bid/Bax ↑ ER stress responses	↓ Tumour cell growth ↓ Cellular proliferation	Apoptosis	[18]
Breast	Receptor- Independent	↓ AKT and mTOR signalling ↑ Reactive oxygen species (ROS) ↑ ER stress responses	↑ Cytotoxicity ↓ Tumour cell migration	Autophagy Apoptosis	[12]
Pancreatic	CB2	↑ Ceramide synthesis ↑ ER stress ↑ p8–TRIB3 induction ↑ AKT inhibition	↑ Antitumor action ↓ Tumour cell growth ↓ Cellular proliferation	Autophagy Apoptosis	[19]
Colon	ND	↑ Mitochondrial Ca^2+^ ↑ ER stress responses	↓ Cellular proliferation ↑ Cytotoxicity	ND	[14]
Prostate	ND	↑ Caspase3/7 activity ↓ RBBP6 ↑ p53; bax mRNA expression ↓ Bcl2 gene expression	↓ Cellular proliferation ↓ Tumour cell growth	Apoptosis	[20]
Prostate	CB1, CB2	↓ PSA, VEGF, ↓ Chemokine IL-6 and IL-8. ↓ CB1, CB2 on cancer cells ↓ Pro-inflammatory cytokines	↑ Anti-inflammatory ↑ Cytotoxicity ↓ Cancer cell growth	Autophagy	[13]
Leukaemia	CB2	↑ ER stress ↑ p8–TRIB3 induction	ND	Autophagy Apoptosis	[21]
Melanoma	CB2	↑ p8–TRIB3 induction ↓ AKT and mTOR signalling	↓ Cell growth ↓ Cell viability, ↓ Invasion ↓ Metastasis ↓ Proliferation	Autophagy Apoptosis	[22]

↑ Upregulation/increase; ↓ downregulation/decrease; ND = not determined.

## Data Availability

Not appliable.
